# Endoscopic transmural drainage is associated with improved outcomes in disconnected pancreatic duct syndrome: a systematic review and meta-analysis

**DOI:** 10.1186/s12876-021-01663-2

**Published:** 2021-02-25

**Authors:** Eric Chong, Chathura Bathiya Ratnayake, Samantha Saikia, Manu Nayar, Kofi Oppong, Jeremy J. French, John A. Windsor, Sanjay Pandanaboyana

**Affiliations:** 1 Surgical and Translational Research Centre, Faculty of Medical and Health Sciences, University of Auckland, Auckland, New Zealand; 2 Department of Radiology, Freeman Hospital, Newcastle upon Tyne, UK; 3 Department of Gastroenterology, Freeman Hospital, Newcastle upon Tyne, UK; 4 HPB and Transplant Unit, Department of Hepatobiliary, Pancreatic and Transplant Surgery, Freeman Hospital, Newcastle upon Tyne, UK; 5 Population Health Sciences, Newcastle University, Newcastle upon Tyne, UK

**Keywords:** Disconnected pancreatic duct, Pancreatic duct disruption, Acute necrotizing pancreatitis, Pancreatic fistula

## Abstract

**Background:**

Disconnected pancreatic duct syndrome (DPDS) is a complication of acute necrotizing pancreatitis in the neck and body of the pancreas often manifesting as persistent pancreatic fluid collection (PFC) or external pancreatic fistula (EPF). This systematic review and pairwise meta-analysis aimed to review the definitions, clinical presentation, intervention, and outcomes for DPDS.

**Methods:**

The PubMed, EMBASE, MEDLINE, and SCOPUS databases were systematically searched until February 2020 using the PRISMA framework. A meta-analysis was performed to assess the success rates of endoscopic and surgical interventions for the treatment of DPDS. Success of DPDS treatment was defined as long-term resolution of symptoms without recurrence of PFC, EPF, or pancreatic ascites.

**Results:**

Thirty studies were included in the quantitative analysis comprising 1355 patients. Acute pancreatitis was the most common etiology (95.3%, 936/982), followed by chronic pancreatitis (3.1%, 30/982). DPDS commonly presented with PFC (83.2%, 948/1140) and EPF (13.4%, 153/1140). There was significant heterogeneity in the definition of DPDS in the literature. Weighted success rate of endoscopic transmural drainage (90.6%, 95%-CI 81.0–95.6%) was significantly higher than transpapillary drainage (58.5%, 95%-CI 36.7–77.4). Pairwise meta-analysis showed comparable success rates between endoscopic and surgical intervention, which were 82% (weighted 95%-CI 68.6–90.5) and 87.4% (95%-CI 81.2–91.8), respectively (*P* = 0.389).

**Conclusions:**

Endoscopic transmural drainage was superior to transpapillary drainage for the management of DPDS. Endoscopic and surgical interventions had comparable success rates. The significant variability in the definitions and treatment strategies for DPDS warrant standardisation for further research.

## Background

Disconnected pancreatic duct syndrome (DPDS) complicates 30% of patients with acute 1necrotizing pancreatitis and commonly manifests as persistent pancreatic fluid collections (PFC) or external pancreatic fistulae (EPF) [1–5]. Pancreatic necrosis involving the main pancreatic duct (MPD) results in the disconnection between the proximal MPD and the distal remnant gland. Therefore, pancreatic secretion from the viable distal remnant gland will not reach the duodenum but will drain to the retroperitoneal and/or retrogastric space to cause PFCs or towards the peritoneum to cause pancreatic ascites. Treatment of PFC with percutaneous or surgical drains run a significant risk of persistent EPF and is therefore no longer recommended. Short of atrophy or definitive treatment, PFC or EPF tends to persist due to continuous secretion from the viable distal remnant gland. Therefore, active diagnosis and management of DPDS is important as its resolution is unlikely with expectant management [1].

A certain diagnosis of DPDS requires the presence of three criteria: a) necrosis of at least 2 cm length of pancreas, b) viable pancreatic tissue upstream from the site of necrosis (ie, toward the pancreatic tail), and c) extravasation of contrast material-injected from the MPD at pancreatography [6]. Although surgery was historically recommended for all DPDS, endoscopic techniques have evolved from endoscopic transpapillary stenting and drainage through to endoscopic ultrasound and endoscopic transmural drainage of DPDS [4, 7–9]. ‘Endoscopic drainage’ for DPDS is now categorized as transpapillary drainage, transmural drainage, or a combination of both techniques.

Selecting the optimal treatment remains challenging as there are few quantitative comparative studies on which to base the decision. A previous review of DPDS found no differences among various endoscopic drainage and surgery techniques [7]. However, that analysis was hampered by heterogeneity in its study populations specifically due to the inclusion of patients with partial duct disruption [7]. This potentially skewed the results to favour endoscopic drainage, especially transpapillary drainage, because partial duct disruptions have significantly better outcomes when treated endoscopically with transpapillary stenting or drainage and were generally not treated with surgery. Therefore, the reported > 80% success rates for endoscopic or surgical interventions had limited generalizability to DPDS treatment.

This systematic review and meta-analysis aimed to review the definition, presentation, intervention and outcomes of DPDS as well as the treatment outcomes for surgery and endoscopic drainage in patients with DPDS.

## Methods

### Study selection

The study was carried out according to the Systematic Reviews and Meta-analysis (PRISMA) guidelines [10]. A systematic literature search was performed in four databases: PubMed, MEDLINE, Embase, and Scopus for studies published up to 10th February 2020. A detailed overview of the search and syntax is presented in the Appendix. The reference lists of studies included for full-text review were further screened to identify additional articles not captured on the initial search and screening process.

### Eligibility criteria

After removal of duplicate studies, the title and abstract of the remaining studies were independently screened by two authors (EC, CBR) for potentially relevant studies. A third author (SP) aided in the resolution of any conflicts by adjudicating disagreements. The inclusion criteria were English studies which reported on complete duct disruption or DPDS in adults (> 18 years) following pancreatitis or trauma. Review articles, opinion statements, editorials, animal studies, case reports, articles including only partial duct disruption, and studies with less than five participants were excluded.

### Critical appraisal

Methodological quality of the studies was independently assessed by two authors (EC, CBR) using the ROBIN-I tool [11]. Any discrepancy was adjudicated by a third author (SP). The overall risk of bias was based on seven domains with assessment guided by signaling questions. The seven domains were related to biases that could arise in nonrandomized studies and were broadly categorized as pre-intervention, at intervention, and post-intervention. The risks of bias of each domain were classified as low, moderate, serious, or critical. The overall risk of bias was based on the domain which had the highest risk of bias and was likewise classified as low, moderate, serious, or critical [11].

### Data extraction

Two authors (EC, CBR) independently performed the data extraction onto a preformed template. Discrepancies in the extracted data were discussed and rationalized and any enduring disagreements were once again adjudicated by a third author (SP). The following study characteristics were extracted: title, authors, year of publication, follow-up duration, type of duct disruption, size of study population, etiology of MPD disruption, and site of disruption. Relevant types of disruption were complete duct disruption and DPDS. Treatment strategies, treatment outcomes, and complications were also extracted.

### Terminology and definitions

DPDS was defined by the evidence of complete discontinuity of the MPD with specific diagnostic criteria outlined in each study and included the term complete duct disruption. Transpapillary drainage referred to the drainage approach via insertion of transpapillary stent into the MPD. Sphincterotomy alone was not considered as transpapillary drainage. Transmural drainage was defined as an endoscopic approach that involved formation of fistula between PFC and the gastrointestinal tract, usually the stomach or duodenum [1]. Plastic double pig-tails stents were usually used to maintain patency of the fistula. In selected cases, metal stent was deployed and on follow-up procedure removed or replaced with double pig-tail stents for long-term drainage [3, 12, 13]. Combined-modality drainage was defined as the combined approach of using transpapillary and transmural drainage. It is a distinct technique from dual-modality drainage described as percutaneous necrosectomy followed by transmural drainage [14, 15]. Surgical drainage referred to surgeries that reestablish drainage of pancreatic secretion into gastrointestinal tract and included Roux-en-Y (RNY) drainage by pancreaticojejunostomy, pancreaticogastrostomy, fistulojejunostomy, cystgastrostomy or cystjejunostomy.

Success was defined as symptoms resolution without recurrence of PFC, ascites, or EPF on long-term follow-ups. The need for additional surgery following endoscopic or surgical intervention was considered failure and thus also determined the number of successes.

### Statistical analysis

RStudio was utilised to perform the statistical analysis through the use of the packages; meta, metafor and tidyverse (R Foundation for Statistical Computing, Austria 2014) [16–18]. Weighted success rates were determined by a random intercept logistic regression when three or more sets of data were available for the analysis. A pairwise meta-analysis was also performed employing a Mantel–Haenszel random effects model and outputs reported by respective odds ratios (OR) with 95% confidence intervals (CI). Statistical heterogeneity was evaluated using I^2^ values whereby a threshold of 25%, 50%, and 75% were indicative of low, moderate, and high heterogeneity, respectively. Heterogeneity was considered nonsignificant when I^2^ < 25% [19]. Studies were further included in the quantitative analysis for a treatment modality if the total number of patients within the relevant treatment arm was three or more. A subgroup analysis was also performed for transmural and transpapillary drainage to compare rates of success.

## Results

### Study characteristics

**Fig. 1:**
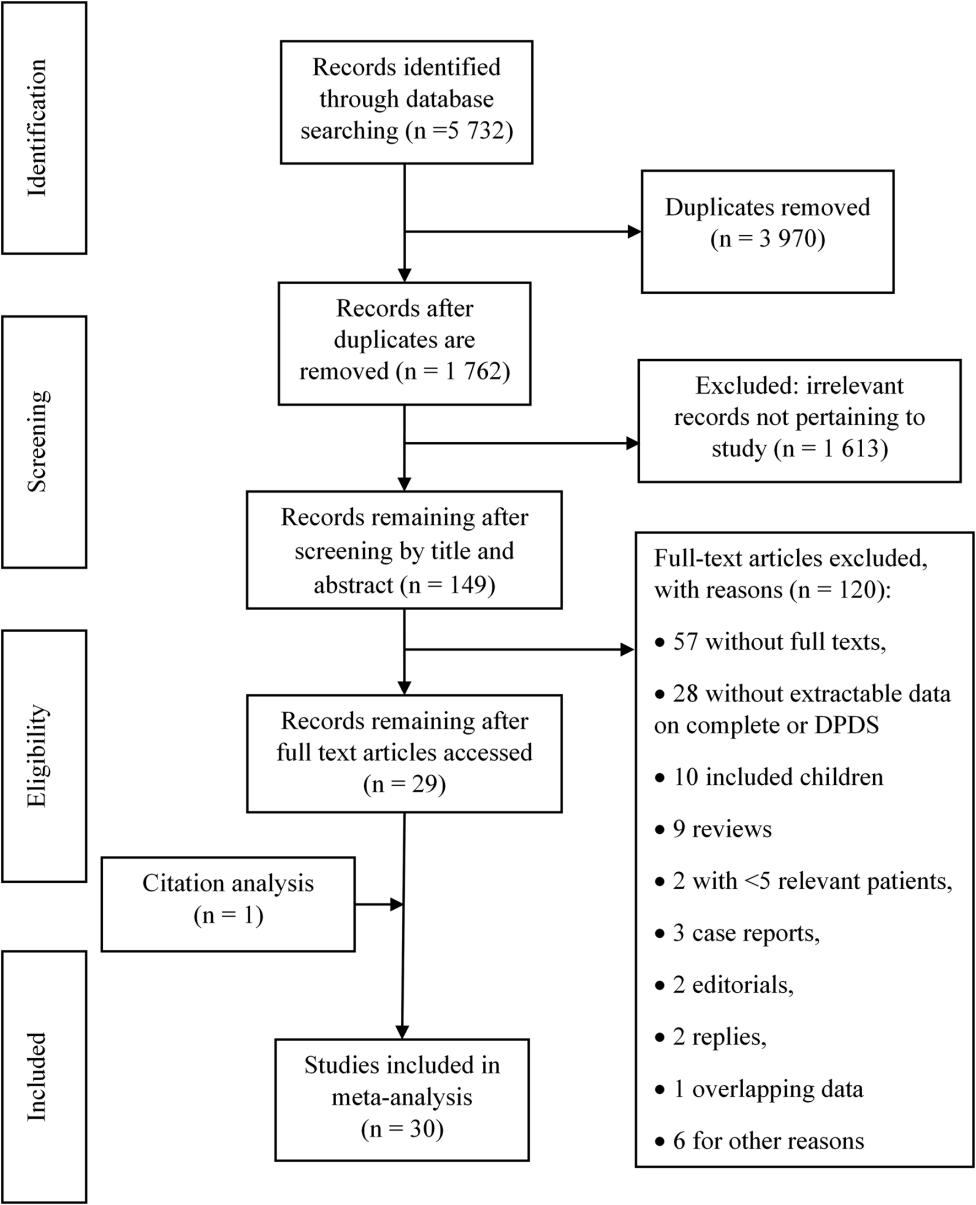
PRISMA flow chart of literature search strategy

The systematic search of databases identified 5 723 articles and included a total of 30 studies in the quantitative systematic review and meta-analysis (Fig. 1). The 30 studies were published between 1995 and 2020 and enrolled 1 355 patients with a diagnosis of DPDS (Table 1). Twenty-seven studies were retrospective in nature [2–5, 8, 12, 13, 20–39], two studies were prospective in design [9, 40], and one study included patients enrolled prospectively and patient data sourced retrospectively [41]. Studies were conducted in the United States (n = 19) [2–5, 8, 9, 13, 21, 24–28, 31, 34–37, 40], India (n = 4) [23, 32, 33, 38], Poland (n = 2) [29, 30], Mexico (n = 1) [41], Belgium (n = 1) [39], China (n = 1) [22], and Japan (n = 1) [20]. One study enrolled patient from India and United States [12].

**Table 1:** Author, year of publication, country, inclusion period, number of included patients and relevant patients, study design, and follow-up interval of included studies

First author	Year of publication	Country	Inclusion period	Included patients	Relevant patients	Study design	Follow-up interval (months)^a^
Devière et al. [39]	1995	Belgium	Jun 1986–Jul 1993	13	13	Retrospective study	28 (0–36)
Howard et al. [9]	2001	United States	June 1995–June 2000	27	27	Prospective study	18
Tann et al. [4]	2003	United States	1995–2000	26	26	Retrospective study	18^c^
Varadarajulu et al. [26]	2005	United States	1994–2002	97	23	Retrospective study	24 (6–86)
Lawrence et al. [2]	2008	United States	Mar 1997–Jun 2003	30	30	Retrospective study	38 (3–94)
Pelaez-Luna et al. [21]	2008	United States	Jan 1999–Jul 2006	31	31	Retrospective study	7 (0–90)
Nealon et al. [8]	2009	United States	1985–2006	563	130	Retrospective study	56.4 ± 12.6^c^
Murage et al. [34]	2010	United States	Nov 1995–Sept 2008	76	76	Retrospective study	22
Varadarajulu et al. [25]	2011	United States	Jan 2003–Apr 2011	62	22	Retrospective study	1026 (678–1036) days^b^
Irani et al. [27]	2012	United States	Oct 2002–Oct 2011	15	15	Three were retrospectively identified patients and other 12 patients were included prospectively	25 (6–113)
Pearson et al. [5]	2012	United States	2002–2011	7	7	Retrospective study	264 (29–740) days
Bang et al. [28]	2013	United States	2003––2011; Jan–Dec 2012	76	53	Retrospective study	309.5 (241.5 -362.5) days^b^
Shrode et al. [31]	2013	United States	Jan 2002–July 2008	113	64	Retrospective study	12^d^
Fischer et al. [35]	2014	United States	Jul 2005–Jun 2011	50	50	Retrospective study	18^c^
Smoczyński et al. [30]	2015	Poland	2001–2013	22	8	Retrospective study	1 year^d^
Rana et al. [33]	2015	India	2010––2014	35	35	Retrospective study	28.2 ± 14.0^c^
Tellez-Avina et al.[41]	2016	Mexico	2008–2015	21	21	Retrospective analysis of prospectively collected data	28 (7–76)
Bang et al. [40]	2016	United States	May 2014–Nov 2015	21	21	Prospective study	272 days
Dhar et al. [24]	2017	United States	2002–2014	42	42	Retrospective study	18
Jagielski et al. [29]	2018	Poland	2001––2016	226	63	Retrospective study	65 (14–158)^c^
Bang et al. [3]	2018	United States	Aug 2003–Dec 2015	291	167	Retrospective study of a prospectively maintained database	1,823 (723–2,656) days^b^
Dua et al. [13]	2018	United States	2009––2017	74	22	Retrospective study	14 (7–27)^c^
Dhir et al. [12]	2018	United States and India	Mar 2011–Dec 2016	88	53	Prospective study	22 (3–46)
Chen et al. [22]	2019	China	Sept 2008–Jan 2016	31	31	Retrospective study on a prospectively maintained database	40 (22–110)
Yamauchi et al. [20]	2019	Japan	Apr 2006–Mar 2017	36	9	Retrospective study	56.2 (12.4–147.1)
Rana et al. (1)^e^ [32]	2019	India	Dec 2011–Nov 2017	12	9	Retrospective study	25.5 ± 17.7 weeks^d^
Rana et al. (2)^e^ [23]	2019	India	2014–2019	18	18	Retrospective study	16.7 ± 12.8^c^
Maatman et al. [36]	2019	United States	2005–2017	202	202	Retrospective study	30 (2–165)
Rana et al. (3)^e^ [38]	2019	India	2015–2019	46	33	Retrospective study of a prospectively maintained database	32.5 ± 21.9
Maatman et al. [37]	2020	United States	2005–2018	714	54	Retrospective study	17.9 (3–150)

^a^Reported by median (range) if provided, or

^b^By median (interquartile range) if provided, or

^c^By mean (range) if provided

^d^Reported as unspecified average (mean or median) by study

^e^Three studies with the same first author and year of publication were denoted with (1), (2), and (3) here and in subsequent tables and figures for clarity

### Presentation and diagnosis

The overall median age of the included cohort was 52 years (range 36–61) [2–5, 21, 23, 24, 27, 29, 32–35, 37–41] with a higher male proportion (1.75:1) [2, 3, 9, 21–24, 27, 29, 32–35, 37–41]. PFC was the most common presentation for DPDS (83.2%, 948/1140) followed by EPF (13.4%, 153/1140), recurrent pancreatitis (2.6%, 30/1140), and ascites (0.8%, 30/1140) [3–5, 8, 9, 12, 13, 20–25, 27–30, 33]. Presentation of DPDS was not quantified or reported in 6 studies [2, 26, 31, 34, 35]. The total prevalence of walled-of necrosis (WON) and pseudocyst were 65.3% (560/857) and 34.7% (297/857) respectively in 16 studies reporting the type of PFC [3, 4, 8, 12, 20, 24, 28–30, 33, 36, 37, 39–41]. Acute pancreatitis (95.3%, 936/982) was the most common etiology for DPDS, followed by chronic pancreatitis (3.1%, 30/982) and trauma (1.6%, 16/982) [2–5, 9, 13, 20–25, 27, 32–41]. A specific etiology of DPDS was not reported in seven studies [8, 12, 26, 28–31]. The most common etiology for acute pancreatitis resulting in DPDS was gallstones (41.7%, 354/848) [2–4, 9, 21–25, 27, 32–36, 38, 40, 41], followed by alcohol (27.2%, 231/848) [2–4, 9, 23–25, 27, 32–36, 38, 40, 41], and idiopathic (12.5%, 106/848) [2, 9, 24, 25, 27, 32–36, 38, 40, 41]. An etiology of acute pancreatitis was not specified in 12 studies [5, 8, 12, 13, 20, 26, 28–31, 37, 39].

The most common site of DPDS was the body of pancreas (47.0%, 117/249). DPDS at the neck (26.5%, 66/249) and head (21.3%, 53/249) of pancreas were also frequently observed [2, 9, 25, 29, 35, 38, 40]. The specific location was not reported in 21 studies (Additional file 1: Table 2) [3, 5, 9, 12, 13, 20, 22–24, 26–28, 30–37, 41].

Twenty-four studies reported a definition for DPDS [2–5, 9, 12, 13, 21–24, 26, 27, 30–32, 34–41]. There were only four studies [13, 34, 36, 37] that used the three criteria proposed by Sandrasegaran et al. [6]. Seven studies defined DPDS by two criteria, which were extravasation or cutoff appearance of MPD when injected with contrast material and the demonstration of a viable upstream pancreas on imaging [2, 3, 5, 27, 29, 40, 41]. Three studies required an additional criterion that was nonhealing EPF or PFC [9, 21, 24]. Eight studies (including five studies on complete duct disruption) used the aforementioned MPD appearance as the sole criterion to define DPDS [22, 23, 29–32, 38, 39]. Lastly, two studies used intraoperative findings to define DPDS, however the specific findings were not reported (Additional file 1: Table 2) [4, 35].

### Endoscopic drainage

**Fig. 2:**
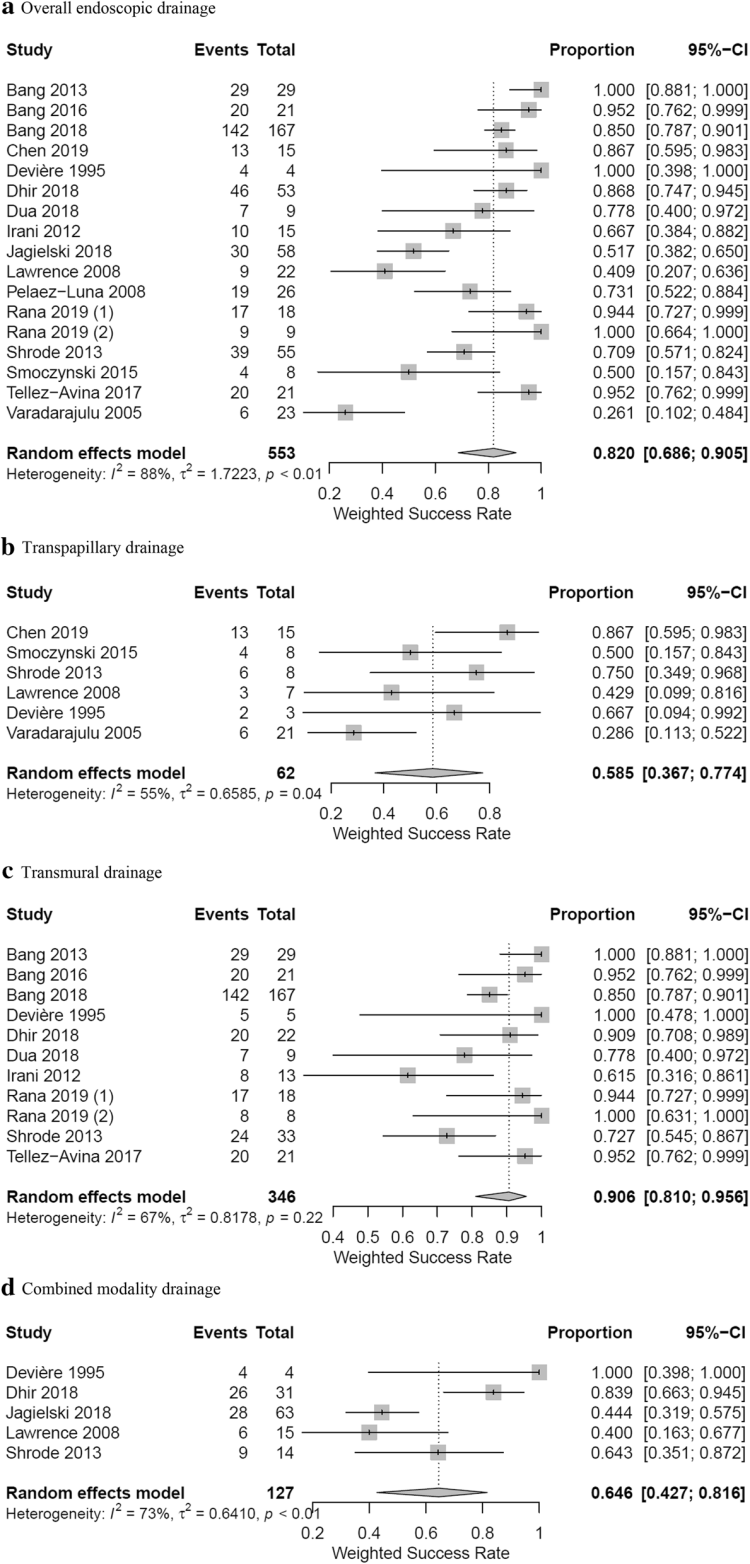
Weighted rates of success of **a** overall endoscopic drainage, **b** transpapillary drainage, **c** transmural drainage, and **d** combined-modality drainage

The average time between the onset of pancreatitis, EPF, or PFC and diagnosis of DPDS was between 56 days and 7.5 months in two studies [22, 23]. 17 studies reported on endoscopic drainage of DPDS including transmural, transpapillary, and combined-modality drainage which included a total of 553 patients [2, 3, 12, 13, 21–23, 26–32, 39–41]. The weighted overall success rate for endoscopic drainage was 82.0% (95%-CI 68.6–90.5%) (Fig. 2a). Six studies reported treatment of a total of 62 patients with transpapillary drainage [2, 22, 26, 30, 31, 39]. The weighted overall success rate in these studies was 58.5% (95%-CI 36.7–77.4%) (Fig. 2b). Eleven studies reported treatment outcome of transmural drainage which included 346 patients [3, 12, 23, 27, 28, 31, 32, 39–41]. Transmural drainage was associated with a weighted success rate of 90.6% (95%-CI 81.0–95.6%) (Fig. 2c). Six of the 11 studies solely performed endoscopic ultrasound (EUS) guided transmural drainage for a total of 84 patients [12, 13, 27, 32, 40, 41]. The weighted success rate of the six studies were 91.7% (95%-CI 83.5–96.0%) (Additional file 1: Fig. 1). Transmural drainage success rate on weighted analysis was significantly higher compared to transpapillary drainage. Nine of the 11 studies reported the duration of transmural stents that were left in-situ [3, 12, 13, 23, 27, 28, 32, 40, 41]. There were five studies that left stents in-situ indefinitely [27, 28, 32, 40, 41], three studies that routinely removed the stents [12, 13, 21], and one study that removed the stents routinely in initial years of practice but later left the stents in-situ indefinitely [3].

Five studies reported treatment of a total of 131 patients with combined-modality drainage [2, 12, 29, 31, 39]. Combined-modality drainage was associated with a weighted success rate of 64.6% (95%-CI 42.7–81.6%) (Fig. 2d). Two of the five studies routinely removed transmural stents for their patients [2, 12]. The other three studies did not report the duration of transmural stent [29, 31, 39].

Ten studies reported the type of transmural stents that were used [2, 3, 12, 13, 23, 27, 28, 32, 40, 41]. Five studies used only double pig-tail stents [20, 23, 27, 28, 41]. Three studies used either solely double pig-tail stents, or metal stents that were later exchanged for double pig-tail stents for long-term drainage [3, 32, 40]. Lastly, two studies exclusively used metal stents for drainage which were routinely removed [12, 13].

Six studies which left transmural stents in-situ indefinitely reported stent-related complications of 19.5% (23/118). All stents left in-situ were double pig-tail stents [20, 23, 25, 27, 33, 41]. All complications were related to stent migrations except for one patient who experienced stent fragmentation and stent migration [27]. Eleven percent (13/118) of the stent-related complications were asymptomatic or incidental findings and 8.5% (10/118) of the complications were symptomatic. These included bowel obstruction (1.7%, 2/118), bowel perforation (1.7%, 2/118), recurrent PFC (1.7%, 2/118), and infection (3.4%, 4/118) [20, 25, 27, 33, 41]. Endoscopic and surgical treatment were needed in 2.5% (3/118) and 0.8% (1/118) of complications respectively [20, 25, 33, 41]. All endoscopic procedures and related outcomes are shown in Additional file 1: Table 3.

### Surgical treatment

**Fig. 3:**
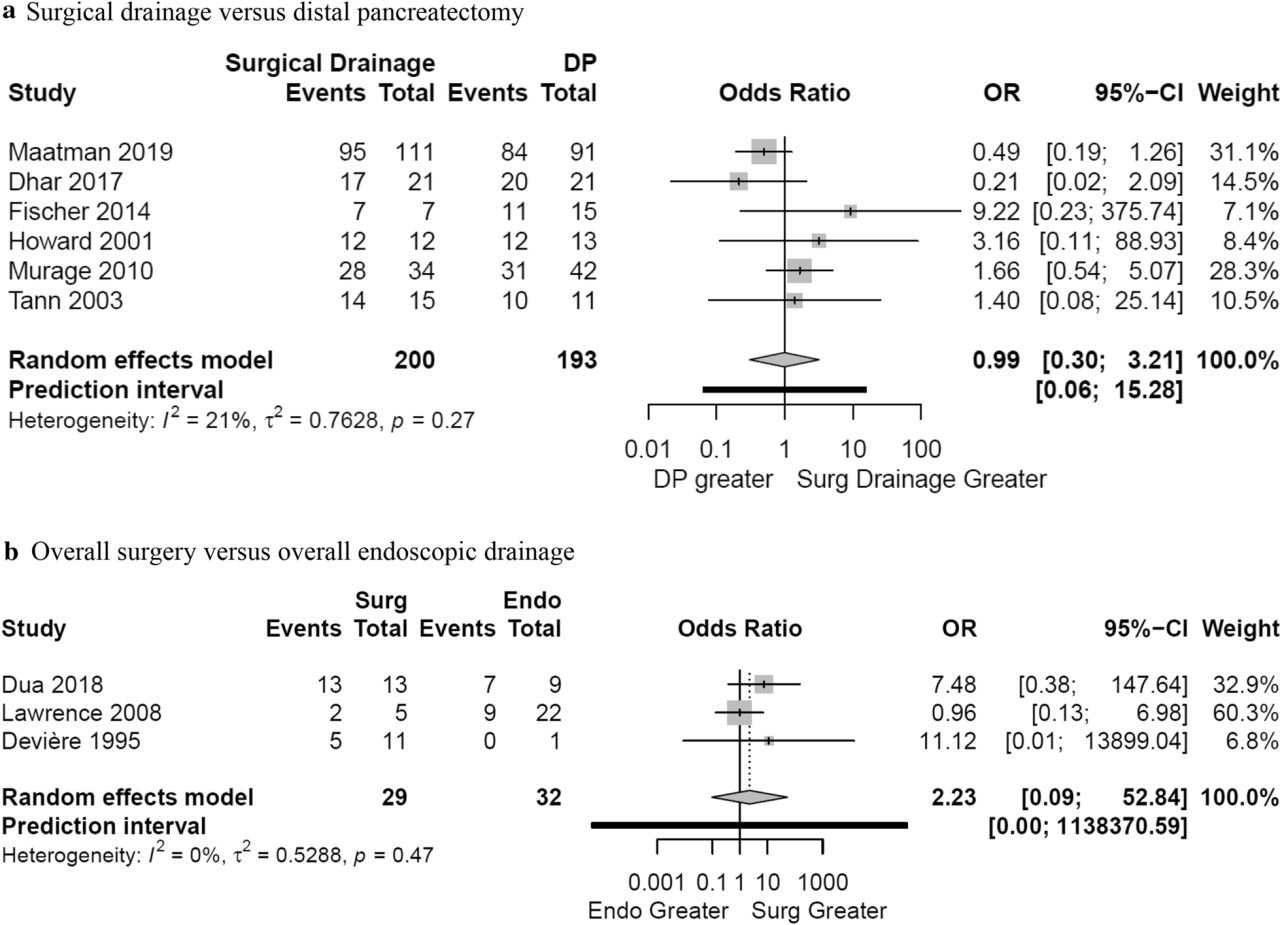
Forest plot of the comparisons **a** distal pancreatectomy vs. surgical drainage and **b** overall surgery vs overall endoscopic drainage. A Mantel–Haenszel random effects model with a Hartung-Knapp adjustment was used for the meta-analysis of all outcomes. A Sidik–Jonkman estimator was utilised for tau. Odds ratios (OR) are shown with 95 percent confidence intervals (CI)

The average time interval between onset of pancreatitis, fluid collection, or fistula and surgery ranged from 3.9 to 6.1 months [4, 5, 9, 35]. Surgery was used as the definitive treatment following failure with endoscopic drainage in 22.0% (84/382) of patients [8, 35, 36]. Ten studies reported on surgical treatment of DPDS including distal pancreatectomy and surgical drainage in 194 and 226 patients respectively [2, 4, 5, 8, 9, 13, 24, 34–36]. The weighted overall success rate for surgical treatment was 87.4% (95%-CI 81.2–91.8%) while the weighted overall success rate of surgical treatment published in the last 10 years was 84.7% (95%-CI 78.7–89.2%) in five studies (Additional file 1: Fig. 2a and b) [5, 13, 24, 35, 36]. The weighted success rate for distal pancreatectomy and surgical drainage was 86.6% (95%-CI 77.0–92.6%) and 85.8% (95%-CI 80.7–89.8%) respectively (Additional file 1: Fig. 2c and d). No difference was observed on pairwise meta-analysis between distal pancreatectomy and surgical drainage (distal pancreatectomy, 86.8%, 168/194, vs. surgical drainage, 86.3%, 195/226, OR 0.99, 95%-CI 0.30–3.21, *P* = 0.981) (Fig. 3a). All surgical procedures, prior therapy, and related outcomes are shown in Additional file 1: Table 4.

There was no difference found between surgical treatment and endoscopic drainage on pairwise meta-analysis (surgery, 69.0%, 20/29 vs. endoscopic drainage, 50%, 16/32, OR 2.23, 95%-CI 0.09–52.84, *P* = 0.389) (Fig. 3b). Similarly, there was no difference found between weighted overall success rates of surgical treatment and endoscopic drainage (Fig. 2a and Additional file 1: Fig. 2a).

### Percutaneous drainage

Five studies reported on percutaneous drainage in 161 patients [2, 8, 31, 37, 38]. Percutaneous drainage was uniformly unsuccessful in three studies [2, 8, 31]. In the other two studies, percutaneous drainage resulted in successful treatment in 97.0% (32/33) and 22.2% (12/54) of patients (Additional file 1: Table 5).

### Quality assessment and heterogeneity

**Fig. 4:**
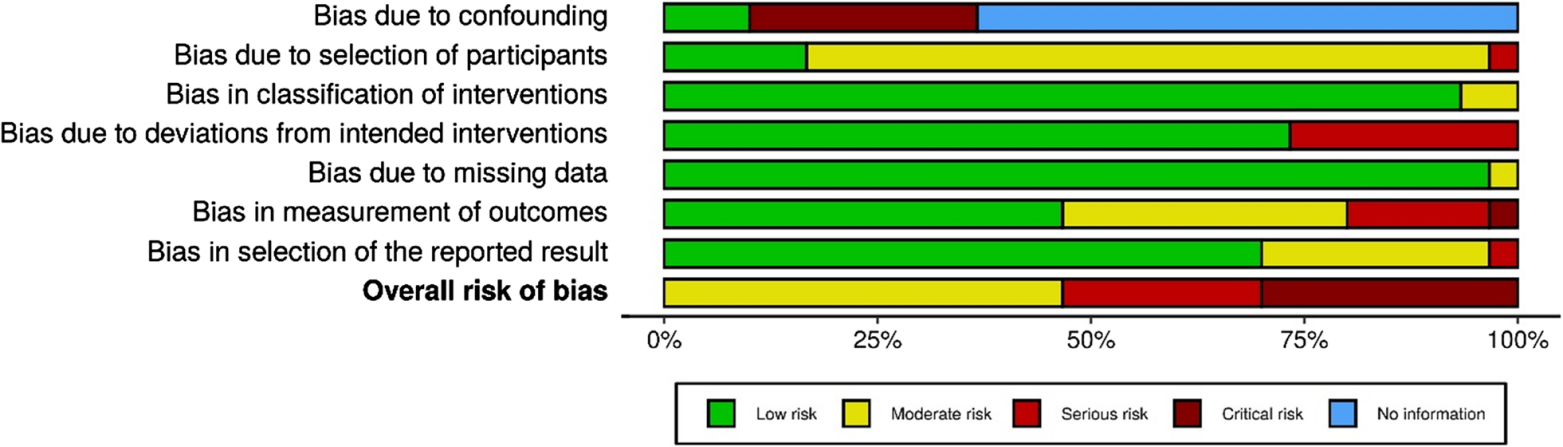
Risk of bias summary: review authors’ judgements about each risk of bias items presented as percentages for each presented study

Quality assessment using the ROBIN-1 tool demonstrated overall risk of bias were moderate in 14 studies [2, 4, 9, 12, 20, 21, 23, 25, 27, 28, 33, 36–38], serious in 7 studies [3, 5, 8, 13, 24, 29, 34], and critical in 9 studies [22, 26, 30–32, 35, 39–41]. The nine studies were at critical overall risk of bias due to significant deficiencies in the domain of confounding bias [22, 26, 30–32, 38, 39, 41]. A detailed quality assessment using the ROBIN-1 tools for individual studies is presented in Additional file 1: Table 1. For an overview of risk of bias items across all studies, see Fig. 4.

Statistical heterogeneity was high (I^2^ > 75%) in weighted analysis of overall endoscopic drainage (I^2^ = 88%) and moderate (I^2^ > 50%) in weighted analyses of transpapillary drainage (I^2^ = 55%), transmural drainage (I^2^ = 67%), combined modality drainage (I^2^ = 73%), and overall surgical treatment (I^2^ = 62%). Examination of the forest plots revealed sources of heterogeneity were largely due to outliers which had success rates markedly lower than the pooled results. Several reasons for lower success rates exist. In weighted analyses of endoscopic interventions, lower success rate in three studies may be attributed to routine removal of stents [2, 13, 31]. In two studies lower success rates may be due to addition of resolution of MPD disruption as a required criterion for therapeutic success [26, 29]. In one study, the study population included only patients with EPF and used complex endoscopic techniques during transmural drainage, which may contribute to its lower success rate [27]. Finally, heterogeneity in overall endoscopic or surgical treatment may in part reflect different success rates of different techniques grouped together. Although a subgroup analysis based on ductal anatomy or DPDS definition would be ideal, such analysis was not performed because of limited data.

## Discussion

This contemporary systematic review of 30 studies and 1355 patients has critically appraised the definitions, presentation, intervention, and outcomes from intervention for DPDS. There was significant variability in definitions of DPDS with only four studies using the three criteria proposed by Sandragesaran et al. [6]. PFC was the most common presentation for DPDS, followed by EPF. The comparison of weighted success rates demonstrated endoscopic transmural drainage was superior to transpapillary drainage. The successful outcomes of endoscopic transmural drainage and surgical interventions (distal pancreatectomy or drainage procedures) were similar at 82% and 87.4% respectively.

A recent review reported higher success rates for transpapillary drainage (81.0%) than those observed in the current review (58.5%), a potential consequence of including the less severe partial duct disruption [26, 31, 42], resulting in comparable outcomes for transpapillary and transmural endoscopic approaches. That study also found comparable success between endoscopic and surgical management and so recommended a step-up model to offer surgical treatment of DPDS following endoscopic failure [7]. In this study cohort of patients diagnosed with DPDS, we found transmural drainage to be superior to transpapillary drainage but comparable to surgical management. Transmural drainage was also associated with a reasonably low complication rate. These factors make transmural drainage an attractive first-line treatment option. Endoscopic drainage aside, two studies found subsets of patients with DPDS that responded to percutaneous drainage, which may have occurred as the result of decreased exocrine output over time [38]. However, treatment outcomes with percutaneous drainage were still generally poor.

The development of the DPDS considerably impacts the clinical course following pancreatitis because it does not respond to conservative management [1, 43]. Delays in the diagnosis of DPDS are common and should be suspected in patients who fail to resolve as expected and especially in those who have had documented necrosis of the pancreas [1, 6, 43]. These patients often have increased abdominal discomfort and early satiety because of gastric compression by the PFC [34, 36]. A failure to diagnose DPDS in the context of a persistent PFC can result in suboptimal external drainage leading to a persistent external fistula, multiple re-interventions and delays in definitive treatment for DPDS, all of which prolongs hospital stay and increases treatment costs [1]. In patients who have a PFC and fail to resolve and who have documented pancreatic necrosis (≥ 2 cm) with viable upstream pancreatic tissue (i.e. the first two Sandrasegaran criteria) [6], further imaging is warranted to determine whether there is disruption of the main pancreatic duct prior to any intervention. A magnetic resonance pancreatogram (MRP) will allow delineation of the ductal anatomy, although endoscopic retrograde pancreatography (ERP) may be required to secure the diagnosis of DPDS.

The present systematic review identified significant variation in the definition of DPDS. Aside from the observation that only 24 studies reported a set of criteria and definitions for DPDS, ten studies used the morphology of ductal anatomy on imaging and ERP or MRP [2, 3, 5, 9, 21, 24, 27, 29, 40, 41], four studies further required evidence of necrosis on imaging [13, 34, 36, 37], and eight studies (including five studies on complete duct disruption) solely defined DPDS by the morphology of ductal anatomy on ERP/MRP alone [22, 23, 26, 30–32, 38, 39]. Eighteen studies omitted the length of necrosis from their definitions of DPDS. In a case series of 46 patients, surgically proven disconnected pancreatic ducts had a length of glandular necrosis of > 2 cm [6]. A shorter segment of necrosis is likely to heal by stricture formation [6]. So, the length of necrosis appears to be an important factor to consider. Eight studies defined DPDS solely by ERP findings. They did not account for the inclusion of patients with atrophied distal remnant gland or viable pancreatic tissue bridging the disrupted site, features that are not consistent with a DPDS diagnosis [6]. This finding of the marked variation in the criteria used to diagnose DPDS makes it very difficult to compare different datasets and is a strong call for the standardisation of the DPDS definition.

In this review, PFC (83.2%) was the most common presentation of DPDS, followed by EPF (13.4%). However, predicting the likelihood of developing a disconnected duct remains difficult. Features of persistence or recurrence of collection or fistula, which characterise this syndrome could improve the estimation of pre-test probability for DPDS, but the present review was limited by the absence of clinical course of DPDS in the included studies. In one study, features that were significantly associated with presence of DPDS were WON and multiple PFCs [3]. This review also found WON (65.3%) to be more common than pseudocyst (34.7%) in patients with PFC. Furthermore, this review found pancreatic duct in the body of pancreas to be the most common site of disruption [1]. However, most studies did not report a specific location of disruption.

Historically, surgery was the preferred treatment of DPDS [4], including both resection of the disconnected segment and surgical drainage to re-establishing drainage of pancreatic exocrine secretions into the gastrointestinal tract [1, 34, 36]. Although the initial experience with endoscopic drainage yielded inferior success rates [4, 8], endoscopic management has become increasingly popular following the introduction of EUS-guided transmural drainage and stenting [39, 44, 45]. More recently, the duration of stent placement has been shown to have a significant impact on the recurrence rates of PFC. Arvanitakis et al. in a randomized controlled trial comparing routine early removal of stents versus long-term transmural stents placement demonstrated that long-term stent placement was associated with lower recurrence rates of PFC [44]. Similarly, several observational studies on DPDS also showed long term stents to be associated with significantly lower recurrence rates than routine early stent removal [3, 28].

In this review, double pig-tail stents were more commonly used compared to metal stents. Drainage with metal stents is potentially more attractive than plastic stents with less risk of stent migration, and a wider fistulous tract between the pancreas bed and the stomach would theoretically reduce the chance of recurrence of PFC [12, 46]. However, previous observations of the two stent types found them comparable without high-quality evidence to favor one over the other [47]. Indeed, meta-analyses on the topic have had variable results with more recent publications favoring metal stents over double pig-tail stents [48, 49]. Nonetheless, these studies reported outcomes for PFC in general. DPDS-specific outcomes are still limited with only two retrospective studies in this review that exclusively reported on DPDS treatment using metal stents. The two studies reported success rates of about 80% [12, 13]. Thus, further studies are warranted to confirm the effectiveness of metal stents in DPDS.

This is the first review to exclusively investigate management and outcomes for patients with DPDS and complete duct disruption, excluding those with partial duct disruption, thereby improving homogeneity and the validity of the findings. Given the breadth of the analysis performed, this review provides an exhaustive summary of the literature with a quantitative assessment of relative efficacy of various forms of management. However, several limitations were identified during the conduct of this review. Although the authors aimed to provide homogeneity in the inclusion of DPDS patients by excluding partial duct disruption, five studies failed to provide a definition. This review included mostly observational non-randomized cohorts with significant deficiencies in study methodology as confirmed by the risk of bias assessments. These deficiencies underline the importance of better study design and higher-powered datasets on which to base future recommendations for management. Lastly, the studies included had patient recruitment extending over a long period (1995–2020), and during which period there have been many improvements in the management of acute pancreatitis. In view of these limitations, the findings of the review require confirmation through large registry data and better designed prospective studies. However, the findings from this review show that there is early evidence to suggest transmural drainage may be superior to transpapillary drainage and equivalent to surgical intervention in selected patients. A multi-disciplinary approach is therefore recommended that may step up therapy and identify candidates that may be amenable to transmural drainage and to offer surgery in those who may be higher risk of failure. Furthermore, following widely accepted standardized definitions of DPDS will aid in patient assessment and translation of research findings to clinical practice.

## Conclusion

This systematic review found the treatment success rate of EUS-guided transmural drainage was the highest among endoscopic drainage techniques and was comparable to surgical treatment. However, there was significant variability in the definition of DPDS, which limits the strength of these conclusions. An international collaborative registry using a standardized definition of DPDS is recommended as the next step in evaluating this specific complication of pancreatitis and to guide future studies and recommendations.

## Supplementary Information

**Additional file 1:** **Supplementary Table 1**. Quality assessment using the ROBIN-1 tool. **Supplementary Table 2**. Presentation, etiology, definition of success and duct disruption, and the site of disruption. **Supplementary Table 3**. The number of patients who underwent endoscopic drainage (total, transpapillary, transmural, and combined modality), duration of transmural stent placement, number of successes for each type of endoscopic drainage and procedural-related complication. **Supplementary Table 4**. The number of relevant patients, the number of each type of surgery that was performed, prior therapy before surgery, umber of successes with each surgical intervention, and procedure-related complications. **Supplementary Table 5**. Presentation, number of interventions, concomitant therapy, duration of drainage, number of successes, and complication of patients treated by percutaneous drainage. **Supplementary Fig 1**. Weighted rates of success for transmural drainage with endoscopic ultrasound (EUS). **Supplementary Fig. 2**. Weighted rates of success for A) overall surgical drainage, B) surgery in the last 10 years, C) distal pancreatectomy, and D) surgical drainage.

## Data Availability

The datasets used and/or analysed during the current study are available from the corresponding author on reasonable request.

## Abbreviations

DPDS: Disconnected pancreatic duct syndrome
PFC: Pancreatic fluid collection
WON: Walled-off necrosis
EPF: External pancreatic fistula
MPD: Main pancreatic duct
RNY: Roux-en-Y
