# Supplementary material for: Endoscopic transmural drainage is associated with improved outcomes in disconnected pancreatic duct syndrome: a systematic review and meta-analysis
Source: BMC Gastroenterol. 2021 Feb 25;21:87. doi: 10.1186/s12876-021-01663-2 (PMC7905849; doi:10.1186/s12876-021-01663-2)
Supplement: Supplementary file 1 — Additional file 1: Supplementary Table 1. Quality assessment using the ROBIN-1 tool. Supplementary Table 2. Presentation, etiology, definition of success and duct disruption, and the site of disruption. Supplementary Table 3. The number of patients who underwent endoscopic drainage (total, transpapillary, transmural, and combined modality), duration of transmural stent placement, number of successes for each type of endoscopic drainage and procedural-related complication. Supplementary Table 4. The number of relevant patients, the number of each type of surgery that was performed, prior therapy before surgery, umber of successes with each surgical intervention, and procedure-related complications. Supplementary Table 5. Presentation, number of interventions, concomitant therapy, duration of drainage, number of successes, and complication of patients treated by percutaneous drainage. Supplementary Fig 1. Weighted rates of success for transmural drainage with endoscopic ultrasound (EUS). Supplementary Fig. 2. Weighted rates of success for A) overall surgical drainage, B) surgery in the last 10 years, C) distal pancreatectomy, and D) surgical drainage. [file 12876_2021_1663_MOESM1_ESM.docx]

**Supplementary Table 1: Quality assessment using the ROBIN-1 tool**

**-
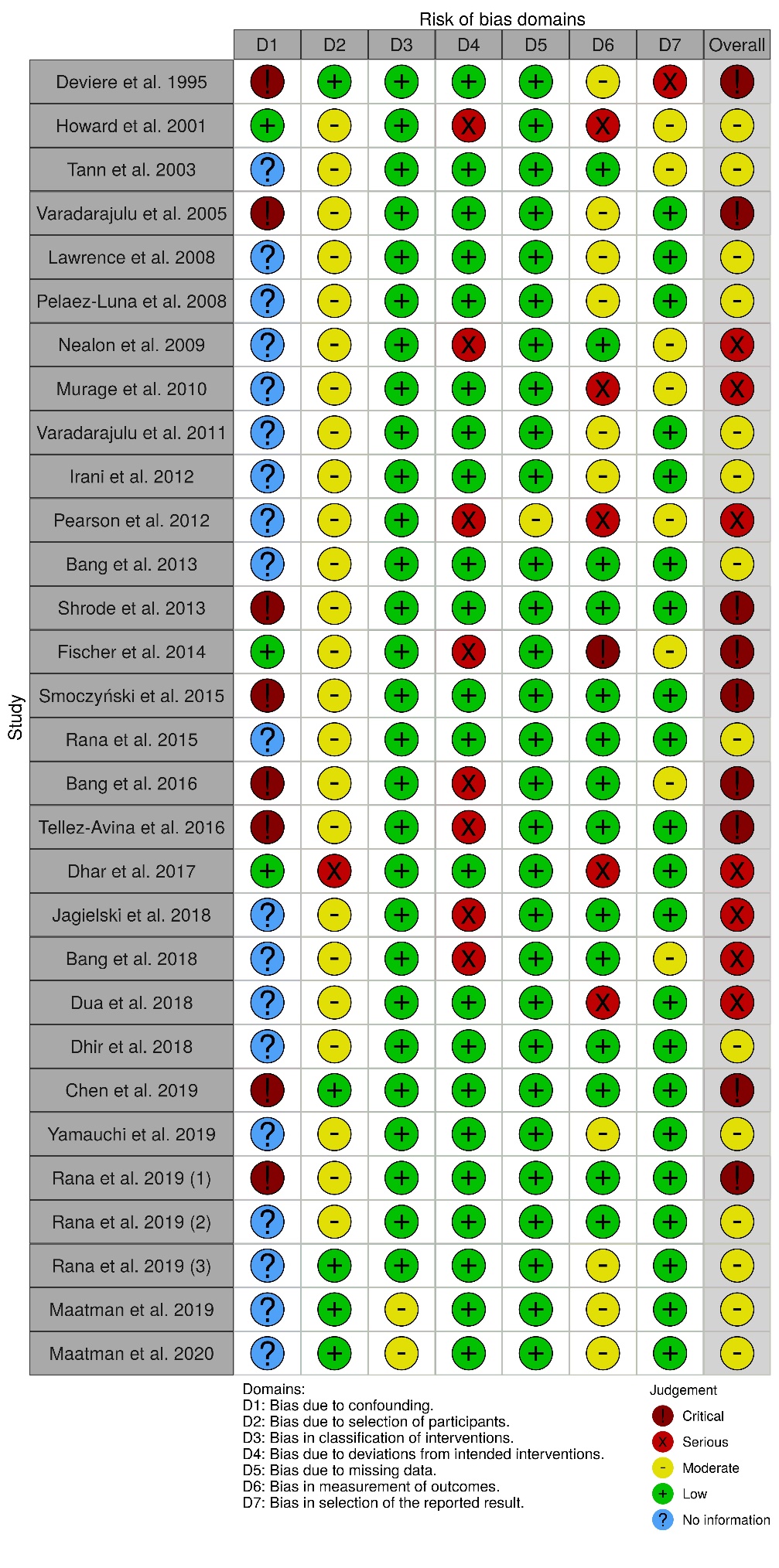
**

**Supplementary Table 2**

Presentation, etiology, definition of success and duct disruption, and the site of disruption

| **Study** | **Relevant patients** | **Manifestation** | **Definition of success by the study** | **Aetiology** | **Site of disruption** | **Operative definition** |
| --- | --- | --- | --- | --- | --- | --- |
| Devière et al. (1995) | 13 complete duct disruption | 12 PSC | NR | AP (n = 9)  CP (n = 4) | 10 proximal (4 head, 6 neck),  3 distal (3 body) | Complete disruption of the MPD was defined as a disruption of the Wirsung duct identified at ERCP and characterized by loss of ductal continuity (of MPD) leading to pseudocyst formation or pancreatic ascites. |
| Howard et al. (2001) | 27 DPDS | 19 EPF, 8 PSC | Complete resolution of fluid collections or fistulae on long-term follow-up | AP (n = 27) | NR | Anatomic criteria for disconnected duct syndrome include (1) ERCP evidence of main pancreatic duct cutoff or discontinuity with inability to access or cannulate the upstream pancreatic duct, (2) computed (CT) evidence of viable pancreatic tissue upstream from the pancreatic duct cutoff or discontinuity, and (3) a nonhealing pancreatic fistula or pseudocyst despite a course of conservative medical management. |
| Tann et al. (2003) | 26 DPDS | 26 PFC | No PFC recurrence | CP (35%)  AP (65%) | 15 proximal (15 neck)  11 distal (5 body, 6 tail) | Operatively defined DPDS |
| Varadarajulu et al. (2005) | 23 complete duct disruption | PFC, fistula, and pancreatic ascites | Therapeutic success was defined as clinical resolution of ascites, a fluid collection and/or fistula, and resolution of the PD disruption at the final ERCP. Therapeutic failure was defined as persistence of the PD disruption, ascites, fluid collection, or fistula as demonstrated by radiological imaging or repeat ERCP, or the need for surgical/radiological intervention. The outcome was termed indeterminate if there was clinical improvement but pancreatography demonstrated an abrupt PD cutoff or if there were persistent symptoms that warranted further intervention(s), despite documented resolution of the PD leak by pancreatography. Recurrence was defined as documentations of imaging studies of the same type of PFC in the same location or a new PFC in a different location, together with recurrence of symptoms. | Most common etiology was CP | NR | By pancreatography, a PD disruption was defined as ‘‘complete’’ when the main duct proximal to the disruption was not opacified |
| Lawrence, et al. (2008) | 30 DPDS | PFC, EPF, or ascites | No PFC recurrence | ANP (n = 30) | 13 proximal (13 head)  17 distal (14 body, 3 tail) | We defined the DPTS as *both* (a) complete disruption of the pancreatic duct, negative opafication of or guidewire access to the pancreatic duct upstream from the disruption, and (b) CT with contrast-enhancing, viable pancreatic tissue upstream from the point of disruption. |
| Pelaez-Luna et al. (2008) | 31 DPDS | 31 PFC | Endoscopic success was considered when the fluid collections and the clinical parameters completely resolved during treatment.  Endoscopic failure was considered when fluid collection persisted and/or the patient experienced either no clinical improvement or deterioration. | AP (n = 31) | 20 proximal (2 head, 18 neck)  11 distal (8 body, 3 tail) | ERCP evidence of main pancreatic duct cutoff or discontinuity with inability to access or cannulate the upstream pancreatic duct; CT evidence of viable pancreatic tissue upstream from the pancreatic duct cutoff or discontinuity; and nonhealing pancreatic fistula or pseudocyst despite a course of conservative medical management. |
| Nealon et al. (2009) | 130 DPDS | 130 PSC | NR | AP and CP | NR | NR |
| Murage, et al. (2010) | 76 DPDS | NR | No symptomatic recurrence of pancreatic pseudocyst. | SAP (n = 59)  CP (n = 17) | NR | DLPR was defined as follows: (1) necrosis at least 2 cm of pancreas; (2) viable pancreatic  tissue upstream (ie, toward the spleen) from the necrosis; and (3) extravasation of the pancreatic duct contrast material or complete cutoff of the pancreatic duct when traced from the duodenum. |
| Varadarajulu et al. (2011) | 22 DPDS | 22 WON | NR | AP (n = 22) | 6 proximal (6 head)  16 distal (16 body/tail) | NR |
| Irani et al.  (2012) | 15 complete duct disruption | 15 EPF | Successful closure of the EPF was defined by the ability to remove the percutaneous drain without any further pancreatic juice draining to the skin, and the time to closure was defined as the days required to achieve success.  A recurrent PFC was defined as formation of a fluid collection after removal of the percutaneous drain. | AP (n = 15) | NR | DPDS was defined as cutoff or blowout of the pancreatic duct, with the inability to demonstrate upstream the body/tail of the pancreas on a pancreatogram. The upstream disconnected body/tail of the pancreas was the source of EPF in all cases and was identified at fistulogram or MRCP. |
| Pearson et al. (2012) | 7 DPDS | 7 EPF | Initial internalization of EPF and no recurrence of pancreatic fluid collection. | AP (n = 7) | 4 head/neck region  3 body/tail region | All patients received preoperative CT scan of the abdomen and DPDS was confirmed with ERCP or MRCP |
| Bang et al. (2013) | 53 DPDS | 53 WON | Treatment success was defined as complete resolution or  decrease in the size of the PFC to less than 2 cm on CT scan, accompanied by resolution of symptoms at 8-week follow-up.  Treatment failure was defined as persistence or worsening of symptoms in association with PFC that had increased in size or was greater than 2 cm in size on follow-up CT at 8 weeks or a need for surgical intervention.  PFC recurrence was defined as symptomatic peripancreatic fluid collection diagnosed on CT imaging following initial treatment success. | AP and CP | NR | NR |
| Shrode et al. | 64 complete duct disruption | NR | Therapeutic success was defined by resolution of the PD leak and  associated complications (i.e., pancreatic ascites, pseudocyst, pancreaticopleural fistula or poorly organized fluid collection).  If the PD leak has not resolved after 12 months, the patient was considered to have failed therapy. Patients that required surgery prior to resolution were also categorized as treatment failure. | NR | NR | Complete ductal disruption was defined as extravasation of  contrast from the PD without opafication of the proximal PD. |
| Fischer et al. (2014) | 50 DPDS | NR | No PFC recurrence | AP (n = 50) | 14 proximal (14 neck)  14 distal (14 body) | CT images and operative reports of each patient were reviewed by the senior author to ensure that image and operative findings confirmed the presence of a disconnected pancreatic duct. |
| Smoczyński et al. (2015) | 8 duct disruptions: DPDS (n=2), complete duct disruption (n=6) | 8 WON | Long-term success was defined as a lack of clinical symptoms, lack of contrast extravasation from the PD, and size of the collection <3 cm in the abdominal contrast-enhanced computed tomography (CECT) during a 1-year follow-up from the end of active drainage. | NR | NR | Complete PD disruption is a leak of contrast medium from the PD with no visualization of the PD upstream to the leak. |
| Rana et al.  (2015) | 35 DPDS | 35 WON | NR | ANP (n = 35) | NR | NR |
| Bang et al.  (2016) | 21 DPDS | 21 WON | No PFC recurrence | AP (n = 21) | 9 proximal (9 neck)  12 distal (10 body, 2 body-tail) | DPDS by EUS was defined by the presence of a well-defined fluid collection along the course of the main pancreatic duct with the upstream pancreatic parenchyma and duct terminating into the fluid collection.  Subsequently, DPDS was confirmed by ERCP, EUS-guided pancreatogram of upstream pancreas, or surgery. |
| Tellez-Avina et al.  (2016) | 21 DPDS | 12 WON (1 infected WON), 6 PSC (1 infected), 3 pancreatic abscesses | Clinical success was considered as the absence of symptoms and no evidence of collections in a period of time of at least 6 months.  Failure of endoscopic treatment was defined as worsening or persistence of symptoms with evidence of an increase or persistence (> 2cm after 6 to 8 weeks) of the collection.  Recurrence was defined as the presence of collection detected by CT after successful endoscopic treatment. | 18/21(85.7%) had AP | NR | We defined DPDS as the total disruption of the pancreatic main duct with viable pancreatic tissue without connection to the digestive tract. |
| Dhar et al. (2017) | 42 DPDS | 42 WON | Failure was defined as recurrent peripancreatic fluid collections or pancreatic fistulae resulting from persistent pancreatic disruption. | AP (n = 42) | NR | Criteria for pancreatic duct disruption included evidence of duct discontinuity on ERCP or MRCP, viable pancreatic parenchyma distal do discontinuity on CT or MRI, or persistent pancreatic fluid collection or fistula with enzyme-rich fluid. |
| Jagielski et al. (2018) | 63 duct disruptions: DPDS (n=28), complete duct disruption (n=35) | 63 WON | Therapeutic success of WON endotherapy was defined as the absence of symptoms and complete regression of the collection, or collection size <40 mm.  The success of endotherapy of MPD disruption was defined as the lack for contrast flow outside the MPD in patients with WON treated endoscopically for MPD disruption within 24 months from the beginning of endotherapy.  Recurrence of pancreatic fluid collections (PFCs) was defined as collection size >40 mm in imaging examinations, or recurrence of symptoms during follow-up.  Long-term success of WON treatment was defined as the therapeutic success of WON endotherapy, the success of endotherapy of MPD disruption, and the lack of recurrence of PFCs. | AP and CP | 28 proximal (28 head)  35 distal (34 Body, 1 tail) | Complete disruption of the MPD was the flow of contrast outside the duct without contrast filling of the distal part of the MPD.  Disconnected duct syndrome – pancreatic fragmentation – was diagnosed in patients with disruption of the MPD or contrast-filled segment of the MPD, without contrast flow outside the duct in ERP, who had the presence of a distal fragment of the pancreatic parenchyma in imaging examinations |
| Bang et al. (2018) | 167 DPDS | PFC including 9(5.4%) acute collections, 44 (26.3%) PSC, 114 (68.3%) WON | Treatment success was defined as complete resolution or  decrease in the size of the PFC to less than 2 cm on CT scan, accompanied by resolution of symptoms at 8-week follow-up.  Treatment failure was defined as persistence or worsening of symptoms in association with PFC that had increased in size or was greater than 2 cm in size on follow-up CT at 8 weeks or a need for surgical intervention. | AP (n = 167) | 37 head/uncinate  130 body/tail | This was defined as complete disruption of the main pancreatic  duct resulting in a variable portion of the upstream pancreas  becoming isolated from the main pancreatic duct downstream. |
| Dua et al. (2018) | 22 DLPR | 22 WON | Failure is defined as the need for additional surgical treatment following index procedures. | AP (n = 22) | NR | DLPR has previously been defined as having the following  criteria: a section of the pancreatic parenchyma with necrosis of at least 2 cm, viable pancreatic tissue upstream (toward the spleen) based on cross-sectional imaging, and extravasation of pancreatic duct contrast material or complete cutoff of the pancreatic duct when followed to the duodenum on ERCP or MRCP. |
| Dhir et al.  (2018) | 53 DPDS | 53 WON | No PFC recurrence.  Recurrence was defined as occurrence of a fresh PFC after removal of BFMS. | NR | NR | NR |
| Chen et al. (2019) | 31 DPDS | 15 PFCs, 16 EPFs | Clinical success was defined as the absence of symptoms and a continuous decrease in cutaneous leaks or in the size of PFCs after the initial procedure.  Clinical failure was defined as persistent or recurrent PFCs or EPFs or no clinical improvement and/or obvious deterioration. | AP (n = 15). Trauma (n = 16) | 22 proximal (head or neck)  9 distal (body or tail) | We defined DPDS as  total disruption of the MPD such that a guidewire could not traverse this disconnection, with non-opacification of the PD upstream from the site of disruption |
| Yamauchi et al. (2019) | 9 DPDS | 9 WON | NR | AP (n=9) | NR | NR |
| Rana et al. (2019) (1) | 9 complete duct disruption | 8 Pancreatic ascites + PFC,  1 pancreatic ascites only | Treatment success was defined as resolution of symptoms with resolution of pancreatic ascites as well as any associated PFCs on CT, with no need for surgery. | AP (n = 9) | NR | It was defined as complete when the main duct upstream to the disruption was not opacified.  An abrupt cut-off value of the MPD on pancreatogram with inability to traverse the guide wire across was suggestive of DPDS |
| Rana et al. (2019) (2) | 18 DPDS | 18 EPF | Treatment success: defined as successful closure of EPF with removal of PCD catheter and complete cessation of drainage of pancreatic juice from cutaneous site without resorting to surgery.  Treatment failure: Need for surgery either for any complication of EUS guided drainage procedure or non-resolution of EPF. | AP (n = 18) | NR | DPDS was defined as either a cut off or blowout of main PD with diffuse extravasation of contrast along with inability to demonstrate upstream PD on pancreatogram obtained during ERCP. |
| Rana et al. (2019) (3) | 33 DPDS | 33 EPF | Successful was defined as closure of EPF that was defined as closure of EPF with removal of  percutaneous drainage (PCD) and complete cessation of drainage of pancreatic  juice from cutaneous site without resorting to surgery. | AP (n=33) | Neck (n=4)  Body (n=29) | DPDS was defined as either a cutoff or blowout of main PD with inability to demonstrate upstream PD on pancreatogram. On MRCP, DPDS was defined as dilated upstream PD with a disconnected segment of pancreas downstream to dilated PD. |
| Maatman et al. (2019) | 54 DPDS | 54 WON | Definitive management with percutaneous drainage alone (PD)  was achieved if the patient met all of the following criteria: no  additional modality of intervention (endoscopic, surgical) following PD, clinical and radiographic resolution of necrosis (absent  drain output in the setting of resolved collection on cross  sectional imaging), and no repeat intervention or evidence of  recurrent collection on follow-up imaging three months following  the removal of all percutaneous drains | AP (n=54) | NR | Disconnected  pancreatic duct syndrome (DPDS) was diagnosed when necrosis  involved at least 2 cm of pancreas with viable upstream (left-sided)  pancreatic parenchyma and extravasation of contrast or total cutoff  of the main pancreatic duct on cholangiopancreatography |
| Maatman et al. (2020) | 237 DPDS | Recurrent PSC 97(40.9%),  Pancreatic fistula 52(21.9%),  Recurrent acute pancreatitis 30(12.7%),  Necrosis (sterile or infected) 52 (21.9%),  Other 6(2.5%) | Complete resolution of symptoms and PFC/EPF/ascites without recurrence of any collections. | AP (n=226) | NR | Diagnosis was made when the following three criteria were met:  1. Necrosis of at least 2 cm of pancreas;  2. Viable upstream (left-sided) pancreatic tissue;  3. Extravasation of contrast or total cutoff of the main  pancreatic duct on pancreatography |

| **Supplementary Table 3**  The number of patients who underwent endoscopic drainage (total, transpapillary, transmural, and combined modality), duration of transmural stent placement, number of successes for each type of endoscopic drainage and procedural-related complication. | | | | | | | | | | | | |
| --- | --- | --- | --- | --- | --- | --- | --- | --- | --- | --- | --- | --- |
| First author | Relevant  patients | Endoscopic drainage | | | | | Duration of TM  Stent^a^ | Number of Successes | | | | Procedure-related Complication^b^ |
|  |  | Total | TP | TM | CM |  | | Total | TP | TM | CM |  |
| Devière et al. (1995) | 13 | 12 | 3 | 5 | 4 | NR | | 11 | 2 | 5 | 4 | 1 infected PSC following TP stenting, 3 stents migration^s^ |
| Varadarajulu et al. (2005) | 23 | 23 | 21 |  | 2 | NR | | 6 | 6 |  | 0 | NR |
| Pelaez-Luna et al. (2008) | 31 | 26 | 4 | 22 |  | Routine removal | | 19 |  |  |  | NR |
| Lawrence et al. (2008) | 30 | 22 | 7 |  | 15 | Routine removal | | 22 | 3 |  | 6 | 1 mild post-sphincterotomy bleeding |
| Varadarajulu et al. (2011) | 22 | 22 |  | 22 |  | Indefinite | |  |  |  |  | 3 stent migrations:   - 2 presented with SBO (1 needed surgery and the other treated conservatively) - 1 discovered incidentally and removed from PFC cavity |
| Irani et al. (2012) | 15 | 15 | 2 | 13 |  | Indefinite | | 15 | 2 | 8 |  | Adverse events:   - 1 post-procedural fever - 5 TM stents migrations and 1 stent fragmentation in 4 patients led to recurrent PSC with infection in 3 patients but eventless in 1 patient. |
| Bang et al. (2013) | 42 | 29 |  | 29 |  | Indefinite | | 29 |  | 29 |  | 3 TM stent migrations:   - 2 stent migrations leading to SBO (1 treated conservatively. 1 needed surgery). - 1 asymptomatic migration into necrotic cavity |
| Shrode et al. (2013) | 64 | 55 | 8 | 33 | 14 | NR | | 39 | 6 | 24 | 9 | NR |
| Smoczyński et al. (2015) | 8 | 8 | 8 |  |  |  | | 4 | 4 |  |  | NR |
| Rana et al. (2015) | 35 | 35 |  | 35 |  | Indefinite | |  |  |  |  | 8 spontaneous stent migrations which was asymptomatic and without PFC recurrence in 5 patients and led to PFC recurrence in 3 patients (1/3 needed surgery and 2/3 remained asymptomatic despite having PFC) |
| Tellez-Avina et al. (2017) | 21 | 21 |  | 21 |  | Indefinite | | 20 |  | 20 |  | 5(23.8%) complications:   - 2(9%) asymptomatic stent migration. - 1(4.7%) stent migration with suspicion of perforation not confirmed surgically. - 1(4.7%) infection following stent migration - 1(4.7%) infection after drainage. |
| Bang et al. (2016) | 21 | 21 |  | 21 |  | Indefinite | | 20 |  | 20 |  | No major disease-related symptoms were reported on follow-up. |
| Jagielski et al.(2018) | 63 | 58 |  |  | 58 | NR | | 30 |  | 30 |  | NR |
| Bang et al. (2018) | 167 | 167 |  | 167 |  | Indefinite (n=121); routine removal (n=46) | | 142 |  | 142 |  | 6 deaths, 11 adverse events (specific adverse events were NR) |
| Dhir et al. (2018) | 53 | 53 |  | 22 | 31 | Routine removal | | 46 |  | 20 | 26 | NR |
| Rana et al. (1) (2019) | 9 | 9 | 1 | 8 |  | Indefinite | | 9 | 1 | 8 |  | 1 transient fever |
| Chen et al. (2019) | 31 | 15 | 15 |  |  |  | | 13 | 13 |  |  | 1 infected pseudocyst |
| Rana et al. (2) (2019) | 18 | 18 |  | 18 |  | Indefinite | | 17 |  | 17 |  | Immediate: 1 transient abdominal pain,  Late: 5 asymptomatic stent migration |
| Dua et al. (2018) | 9 | 9 |  | 9 |  | Routine removal | | 7 |  | 7 |  | NR |
| Yamauchi et al. (2019) | 9 |  | 9 |  |  | Indefinite | |  |  |  |  | 1 asymptomatic migration led to recurrent PSC.  1 stent migration led to colonic perforation |
| ^a^ Reported for studies that used TM drainage. The duration is reported as routine removal if TM stents were removed following resolution of fluid collection or as indefinite if TM stents were intended to left-in situ permanently.  ^b^ Reported only complications that affect relevant population  CM = combined-modality drainage, NR = not reported, PFC = pancreatic fluid collections, PSC = pseudocyst, SBO = small bowel obstruction, TP = transpapillary, TM = transmural. | | | | | | | | | | | | |

| **Supplementary Table 4**  The number of relevant patients, the number of each type of surgery that was performed, prior therapy before surgery, umber of successes with each surgical intervention, and procedure-related complications | | | | | | | | | | | |
| --- | --- | --- | --- | --- | --- | --- | --- | --- | --- | --- | --- |
| First author | Relevant patients | Surgery | | | | Prior therapy | Number of Successes | | | | Procedure-related Complications^a^ |
|  |  | Total | DP | Surgical drainage | Others |  | Total | DP | Surgical drainage | Others |  |
| Howard et al. (2001) | 27 | 27 | 14 | 13 |  | Prior operations:   - 8(62%) in RNY drainage group - 7(50%) in DPS | 24 | 12 | 12 |  | DPS: 2(14%) pancreatic fistula, 1(7%) recurrent PSC, 1(7%) intraabdominal abscess, 2(14%) postoperative diabetes.  RNY: 1(8%) ventral hernia, 2(15%) AND wound infection.  In each group: 1 death, 1 bleeding, and 1 re-operation occurred. |
| Tann et al. (2003) | 26 | 26 | 11 | 15 |  | NR | 24 | 10 | 14 |  | NR |
| Lawrence et al. (2008) | 30 | 5 |  | 5 |  | NR | 2 |  | 2 |  | NR |
| Nealon et al. (2009) | 121 | 121^b^ |  |  |  | 71 patients had PCD | 115 |  |  |  | NR |
| Murage et al. (2010) | 76 | 76 | 42 | 34 |  | 59 received pancreatic debridement and external  drainage. | 59 | 31 | 28 |  | DP vs. RNY: 5(12%) vs. 1(4%) postoperative DM, 7(17%) vvs. 5(15%) postoperative steatorrhea, 5(12%) vs. 3(9%) overall mortality rate. |
| Pearson et al.  (2012) | 7 | 7 |  | 7 |  | Pancreatic drainage or debridement were performed in all patients | 6 |  | 6 |  | Wound infection, transient anastomotic leak, incisional hernia, percutaneous aspiration of post-operative sterile fluid collection, and infectious colitis. |
| Fischer et al.  (2014) | 50 | 50 | 15 | 7 | 28 | 30 required preoperative endoscopic therapy. 12 required preoperative percutaneous drainage | 39 | 11 | 7 | 21 | Pancreatic necrosectomy group: 10 (36%) class II, 0 Class III, 2(8%) Class Iva; 10(36%) exocrine and 16(57%) endocrine insufficiency;  DP group: 4(27%) Clavien-Dindo Class II; 0 Class III, 0 Class IV; 9(60%) endocrine and 11(73%) exocrine insufficiency.  Longitudinal PJ: 4(57%) endocrine and 1(14%) exocrine insufficiency. |
| Dhar et al.  (2017) | 42 | 42 | 21 | 21 |  | All patients who underwent FJ received percutaneous drainage with U-tube | 37 | 20 | 17 |  | FJ: 4(19.1%) worsening endocrine insufficiency, 5(23.8%) worsening exocrine insufficiency^¥^, 10(47.6%) wound infection, 4(19.1%) pancreatic leak, 8(38.1%) recurrent pancreatitis.  DP: 11(52.4%) worsening endocrine insufficiency, 6(28.6%) worsening exocrine insufficiency, 7(33.3%) wound infection, 3(14.3%) pancreatic leak, 8(38.1%) recurrent pancreatitis. |
| Dua et al. (2018) | 13 | 13 |  | 13 |  |  | 13 |  | 13 |  | NR |
| Maatman et al. (2019) | 202 | 202 | 91 | 111 |  |  | 179 | 84 | 95 |  | Resection vs. internal drainage: 15 (16.5%) vs. 7(6.3%) new-organ failure^¥^, 18(19.8%) vs. 14(12.6%) infectious complication, 20(22.0%) vs. 8(7.2%) POPF^¥^, 16(17.6%) vs. 6(5.4%) percutaneous drain use^¥^, 26(28.6%) vs. 13(11.7%) readmission^¥^, 1(1.3%) vs. 2(1.9%) exocrine insufficiency, 9(17.0%) vs. 2(2.2%) endocrine insufficiency^¥^, 26(28.6%) vs. 13(11.7%) readmission, 54(59.3%) vs. 39(35.1%) morbidity, 4(4.4%) vs. 1(0.9%) mortality. |
| ^a^ Reported only complications that affect relevant population  ^b^121 surgeries among which approximately equal number of cyst-jejunostomy, DP, and PJ were performed  ^¥^ significant difference between treatment arms  DPDS = disconnected pancreatic duct syndrome, DM = diabetes mellitus, DP = distal pancreatectomy, DPS = distal pancreatectomy-splenectomy, FJ = fistulojejunostomy, NR = not reported, PJ = pancreatico-jejunostomy, PSC = pseudocyst, RNY = Roux-en-Y reconstruction. | | | | | | | | | | | |

| **Supplementary Table 5**  Presentation, number of interventions, concomitant therapy, duration of drainage, number of successes, and complication of patients treated by percutaneous drainage | | | | | | |
| --- | --- | --- | --- | --- | --- | --- |
| First author | Presentation | Number of percutaneous drainages | Concomitant therapy | Duration of drainage^a^ | Number of success | Complications^b^ |
| Lawrence et al. (2008) | PFC, ascites, or EPF | 1 | NR | NR | 0 | NR |
| Shrode et al. (2008) | PFC, ascites, or EPF | 2 | NR | NR | 0 | NR |
| Nealon et al. (2009) | PSC | 71 | NR | 69.7±13.6 | 0 | 71/71(100%) sepsis, 7171(100%) persistent fistula |
| Rana et al. (3) (2019) | Low-output EPF^c^ | 33 | Given pancreatic enzyme supplement and antibiotics if sepsis. No octreotide or TPN were given. | 88.2± 63.46 | 32 | 0 infective complications, 9(27%) endocrine insufficiency |
| Maatman et al. (2020) | WON | 54 | NR | NR | 12 | NR |
| ^a^ Reported by median (range) in days  ^b^ Reported only complications that affected patients treated by percutaneous drainage  ^c^ Defined as fistula output of <200 ml/d  EPF = external pancreatic fistula, PFC = pancreatic fluid collection, PSC = pseudocysts, TPN = total parenteral nutrition, WON = walled-off necrosis. | | | | | | |

 **Supplementary Fig 1.** Weighted rates of success for transmural drainage with endoscopic ultrasound (EUS).

**Search Syntax**

**Medline**

(Pancreatic Ducts/ or pancreatic duct.mp.) AND (leak*.mp. OR disconnect*.mp. OR disrupt*.mp. OR (Rupture/ or rupture*.mp.))

**Pubmed**

(Pancreatic Ducts/ or pancreatic duct.mp.) AND (leak*.mp. OR disconnect*.mp. OR disrupt*.mp. OR (Rupture/ or rupture*.mp.))

**EMBASE**

(Pancreatic Ducts/ or pancreatic duct.mp.) AND (leak*.mp. OR disconnect*.mp. OR disrupt*.mp. OR (Rupture/ or rupture*.mp.))

**Scopus**

(TITLE-ABS-KEY (pancreatic AND duct*) AND TITLE-ABS-KEY(leak* OR disrupt* OR disconnect* OR rupture*) Limit: Medicine
